# The Symptoms and Treatment of Chronic Suppuration of the Maxillary Antrum
1Read before the Bristol Medico-Chirurgical Society, February 10, 1897.


**Published:** 1897-03

**Authors:** Barclay J. Baron

**Affiliations:** Physician to the Throat and Nose Department of the Bristol General Hospital


### E SYMPTOMS AND TREATMENT OF CHRONIC SUPPURATION OF THE MAXILLARY ANTRUM.[1]

BY

BARCLAY J. BARON, M.B. Edin.,

*Physician to the Throat and Nose Department of the Bristol General Hospital.*

---

object in bringing this subject forward is to indicate the nts in diagnosis and treatment of empyema of the maxillary trum that have guided me in the conduct of a considerable mber of cases of this disease that have come under my obsertion during the past few years. I have nothing new to y, and since the days of Hunter practically nothing new has en said about it; but I am quite sure, from cases that have me under my care, that the condition is very apt to be overoked and the necessary treatment deferred. We know how sential it is to drain an abscess cavity efficiently, and that lay in securing this drainage means prolongation of the aftereatment. This is eminently true of suppuration in the maxillary itrum.

In nearly all my cases the patients have come to me comaining of discharge from the nose; in one, however, severe

[1] Read before the Bristol Medico-Chirurgical Society, February 10, 1897.

pain in the head, which had been treated as "neuralgia," v the symptom that led to my being consulted. The princi subjective symptom then is *nasal discharge*, and this prese certain well-marked and characteristic features which, tal together, put us far on the way to arriving at a diagnosis. Tl are as follows: (1) It is unilateral. This is always observec we set aside those very rare cases of double antral empyei (2) It is pus unmixed with mucus, and it is commonly of cana yellow colour and quite opaque. (3) It is intermittent in flow from the nostril. The time of the day when it is m marked is usually in the morning hours—after rising in morning until midday. During this time the antrum that filled up during the night empties itself; bending the head in washing the face, writing, or stooping in doing the vari duties of the day, facilitates its escape, so that it will often d from the nostril, and several pocket-handkerchiefs will be soil Later in the day there is always some pus coming, but not the same extent as during the period ending with midday. T total amount varies in different patients very much, as is t case with all inefficiently drained abscess cavities, where t rapidity of secretion varies so greatly. (4) It is fœtid. T smell is that of sulphuretted hydrogen, and is not so overpow ing as the stench of atrophic rhinitis (ozœna), or of syphili ulceration and necrosis of bone. It varies very much different patients, and it is observed in some only at tim *e.g.* in the morning hours when the pus first begins to fl It is noticed more by the patient than by his friends, a thus differs greatly from the stench of ozœna, where, ow to the sense of smell being destroyed, the patient does perceive the fœtor. In antral empyema the sense of sn being preserved, the fœtor is a source of much annoyance the patient.

*Pain* is another important subjective symptom. This is ve variable, both in its situation and its amount, and also in bei spontaneously felt or only elicited on examination. It may felt over the antral region and running up to the outer angle the orbit, it may be located over the orbit, or it may be cc plained of over the temple and round the ear. It may be terril slight; it may return at intervals, and thus simulate a :uralgia," due, doubtless, to tension; or it may be absent. If ent, it may be elicited by pressure or percussion over the rum or the side of the nose, or it may not be felt under any umstances.

Leaving the subjective symptoms and coming to the objective s, we gain a great deal of information by examining the nose noting carefully the character, position, and behaviour of pus that is usually found in it. It is canary-coloured and que, it occupies the position of the lower border of the middle inated body; if we wipe it away with a pledget of wool on robe, another drop makes its appearance in the same spot; l it usually smells of sulphuretted hydrogen. This latter uliarity is, in some cases, especially noticed if we press firmly r this region and squeeze it out.

There are two important manœuvres that we ought, at this ge, to carry out. One is to wipe away the pus and ask the ient to place the head on the knees for a few minutes, when vill usually reappear. The other is to place the patient in a umbent position on the *unaffected* side for ten minutes, when it ll reappear if it has previously been expelled. The above aracteristics of the flow of pus in a case otherwise suspicious antral suppuration are almost completely pathognomonic, ce they are only found in their entirety where this accessory us is affected, and thus serve to exclude all the other accessory uses as the source of the discharge. Especially would I lay ess on the behaviour of the flow of pus on altering the sition of the patient, seeing that no other sinus, save maxillary antrum, can empty itself in the positions above cribed.

There are certain other pathological conditions sometimes nd in conjunction with the foregoing essential symptoms, and ich must be mentioned.

1. Pus may be seen to be flowing from above as well as low the middle turbinated body. This indicates disease of e anterior ethmoidal cells, as well as of the antrum, and there probably present *caries*, resulting in breaking down of the thin artition between the infundibulum and the upper part of the antrum. Direct communication is occasionally found betw[illegible] the anterior and posterior ethmoidal cells and the summit [illegible] the antrum; and the partition between the infundibulum [illegible] the antrum may be deficient, and thus the contents of [illegible] frontal sinus might flow into the cavity below.

2. Cleavage of the middle turbinated.

3. Polypus in the nose. This may be quite extensive, bu[illegible] usually only in small amount. The growth may have prece[illegible] the antral mischief, or may have come later. I think it is [illegible] point to be very carefully considered, whether those cases [illegible] nasal polypi with discharge of *pus before any attempts at remo[illegible] have been made* are not usually associated with antral empye[illegible] the latter condition being the source of the purulent dischar[illegible] We ought never to omit examination of the antrum in su[illegible] cases, which are characterised by great obstinacy.

Lastly, there are two other procedures which we may ca[illegible] out:—

1. Exploring the antrum by means of a Lichtwitz's troca[illegible] and cannula, or a curved aspirating needle to which is attach[illegible] a syringe, either through the inferior meatus after cocainisati[illegible] or through the gingivo-labial fold above the second molar toot[illegible] This I have never found it necessary to do.

2. Transillumination by means of a Voltolini electric lam[illegible] placed in the mouth, the tongue being depressed and the patie[illegible] closing the lips tightly. On turning on a sufficiency of curre[illegible] to bring the lamp to its brightest glow, and the room being com[illegible]pletely darkened, the whole face from the mouth to below th[illegible] orbits is usually lighted up and assumes a bright red colour [illegible] the antra be both empty. If there be a solid tumour or a colle[illegible]tion of pus in the antrum, then we get a dark spot spreading [illegible] from the antral region to below the orbit. If on the other ha[illegible] there be a cystic growth in the antrum, we see that side to [illegible] more brightly illuminated than the other. Undoubtedly this [illegible] the rule; but unfortunately there are exceptions met with, an[illegible] thus some of the value of this method of diagnosis is taken awa[illegible] The following are difficulties in its use: (1) A large gelatinou[illegible] polypus in the nose, by causing enhanced brightness, ma[illegible] simulate a cystic tumour in the antrum. When the polypus [illegible]

emoved and blood-clot occupies the nose, the dark patch may simulate antral empyema. Any obstruction in the nasal passages affects the transmission of light through the antrum. (2) In one of my own cases the dark patch was just as noticeable six weeks after operation, when the discharge had practically ceased and the patient was well, as it was before operation when the antrum was full of pus. This was probably due to inflammatory thickening of the mucous membrane in the interior of the cavity. (3) The dark patch has been observed in atrophic rhinitis, and the antrum was opened and found to be quite normal. From this it follows that this method should be carried out *last* in our investigation of a case; and whilst it corroborates other points in favour of a positive diagnosis if we find the dark shadow, I think we ought not to apply this test at the beginning of our examination and thus bias our minds, as it is not *always* trustworthy, although usually so; on the other hand, it ought never to be omitted in a suspicious case.

We find pus coming from the nose in the following pathological conditions: (1) Purulent rhinitis of children. (2) Atrophic rhinitis (ozœna). (3) Syphilis. (4) Rhinolith, or foreign body. (5) Neoplasm. (6) Nasal diphtheria and tubercle. (7) Disease of the accessory sinuses. Out of this group we get a *unilateral* discharge of pus in the following diseases:—

*Syphilis.*—Here we see the evident ulceration, usually formation of a sequestrum and frequently perforation of the hard palate. The stench also is more intolerable to the friends of the patient, and is altogether different from what we meet with in antral disease.

*Rhinolith, or foreign body.*—Here, examination of the nostril and probing renders it easy to come to a conclusion.

*Neoplasm.*—Polypus with purulent discharge preceding operation is often merely an accompaniment of antral empyema. Growth in the antrum, if solid, is usually malignant, and gives us other signs of its nature, such as distension of the cavity, bleeding, &c.

*Disease of the other Accessory Sinuses.*—This is the only condition that presents real difficulty, and we must rely on the following diagnostic points: (1) Anterior ethmoidal cells. The pus is seen to be dripping down over the middle turbinated body from above, and often the probe enables us to discover caries of the bone, with roughening of its surface, &c. (2) Frontal sinus. Whilst acute inflammatory mischief often attacks the frontal sinus, we rarely see chronic pus formation occur, and then only when the duct is blocked, when we get external manifestations over the brow that guide us. Transillumination here is very valuable. (3) Posterior ethmoidal cells and sphenoidal sinus. The discharge of pus from these sinuses is into the naso-pharynx and not into the nose.

Lastly, the effect on the discharge of pus into the nostril of the position of the patient is extremely important to bear in mind. When lying on the unaffected side, or with the head placed on the knees, only the maxillary antrum has its duct so placed as to enable the cavity to empty itself, and thus we can separate disease in this sinus from that occurring in all the others.

It is a matter of great difficulty to arrive at a *positive* diagnosis of suppuration in the antrum; therefore every point must be carefully considered, and in its logical sequence, and it is always a relief to the operator to find a stream of fœtid pus come from the affected nostril after operation and syringing.

Treatment of the condition means securing drainage of the abscess cavity. If there be polypi, hypertrophic rhinitis, deformity of the septum or any obstructive lesion present, we ought to deal with this first, in order to render the orifice from the antrum into the nostril normally patulous. Cleansing and astringent nasal washes and sprays are also useful in this direction. But it is only rarely that spontaneous cure takes place, especially if the trouble is of long standing, and then we must resort to operative treatment to secure drainage. I always use Charters Symonds's antrum drill, which I have had slightly modified by removing the small nut that secures the drill to the handle, as I have found it in the way. I have, in all the cases on which I have operated, drilled through the alveolar border, after removal of a stump by a dentist; and if cocaine be injected, it is an almost painless operation. I think it is a matter of indifference what point we select for drilling, from the first premolar to the second molar; but if I find the first or second molar tooth decayed or gone, I would use that situation and drill vertically through the alveolus into the cavity. If we use the position of either of the premolars, and especially the first one, we ought to incline the drill slightly backwards and upwards. Usually there is very little bone to be gone through; but in one of my cases I found quite a considerable thickness, and had some trouble to bore through it, and this where a sound molar was extracted. In all my operations but one, the teeth in the upper jaw have been very defective; where there is no damaged tooth in the proper situation it is recommended to bore through above the second molar and thus save the teeth intact. After the operation I insert through the wound a Eustachian catheter or some such metal tube, to which is attached a piece of india-rubber tubing, to which is, in turn, attached a syringe. On syringing warm boric lotion through into the antrum, our diagnosis is verified by seeing a stream of fœtid pus pour out of the nostril. In order to keep the wound in the gum open, I get the dentist to make a silver tube which can be clamped to a convenient tooth, and thus remain fixed in the hole made by the drill, and which I tell the patient to remove two or three times daily, to then pass a small tube with indiarubber tubing and syringe attached to it into the antrum, and to syringe warm antiseptic lotion into the cavity. The dentist's apparatus must always be worn until the conclusion of the case, or the soft parts will close.

It does not matter much what antiseptic we select to make the syringing solution, *e.g.*, sulpho-carbolate of zinc, resorcin, hydronaphthol, boracic and carbolic acid, and if the case drag on a long time, as sometimes occurs, nitrate of silver or tincture of iodine. Some patients are well in six to eight weeks, others not for a year or more.

Some operators make a large opening through the canine fossa, and scrape out all polypoid growths that are found in the antrum, and then pack the cavity with antiseptic gauze, in the obstinate cases. This I have never done, though I have seen several cases in the practice of other operators. Others again push a curved Krause's trocar and cannula through the inside wall of the antrum in the inferior meatus, after cocainisation, and syringe through this opening. I have seen cases in which this has been done, but cannot believe that it is usually commendable, as we do not get nearly to the bottom of the cavity in this way. Others use Mikulicz's spear-shaped knife for the same purpose. But, after all, the time-honoured practice of drilling through the alveolar border, or above it, will be found to be the best procedure, and in most patients to be a thoroughly satisfactory one.

---

After the reading of this paper, Mr. W. H. HARSANT expressed his interest in the subject so ably treated by Dr. Baron, and agreed with him in his remarks as to diagnosis and treatment. He had generally observed a carious tooth in these cases, and was inclined to attribute the cause to this source in a large proportion of cases. He generally advised the removal of one of the upper teeth, and the insertion of a small plate with a silver tube attached, so that the patient could remove it daily and freely irrigate the cavity. Some cases were undoubtedly very chronic, but in the large majority a cure was effected in five or six weeks.

